# MicroRNA Expression Profile of Mouse Lung Infected with 2009 Pandemic H1N1 Influenza Virus

**DOI:** 10.1371/journal.pone.0074190

**Published:** 2013-09-16

**Authors:** Zhihao Wu, Rongzhang Hao, Peng Li, Xiaoai Zhang, Nan Liu, Shaofu Qiu, Ligui Wang, Yong Wang, Wenzhong Xue, Kun Liu, Guang Yang, Jiajun Cui, Chuanfu Zhang, Hongbin Song

**Affiliations:** 1 Department of Infectious Disease Control, Institute of Disease Control and Prevention, Academy of Military Medical Sciences, Beijing, P. R. China; 2 State Key Laboratory of Pathogen and Biosecurity, Beijing Institute of Microbiology and Epidemiology, Beijing, P. R. China; Johns Hopkins University - Bloomberg School of Public Health, United States of America

## Abstract

MicroRNAs have been implicated in the regulation of gene expression of various biological processes in a post-transcriptional manner under physiological and pathological conditions including host responses to viral infections. The 2009 pandemic H1N1 influenza virus is an emerging reassortant strain of swine, human and bird influenza virus that can cause mild to severe illness and even death. To further understand the molecular pathogenesis of the 2009 pandemic H1N1 influenza virus, we profiled cellular microRNAs of lungs from BALB/c mice infected with wild-type 2009 pandemic influenza virus A/Beijing/501/2009 (H1N1) (hereafter referred to as BJ501) and mouse-adapted influenza virus A/Puerto Rico/8/1934 (H1N1) (hereafter referred to as PR8) for comparison. Microarray analysis showed both the influenza virus BJ501 and PR8 infection induced strain- and temporal-specific microRNA expression patterns and that their infection caused a group of common and distinct differentially expressed microRNAs. Characteristically, more differentially expressed microRNAs were aroused on day 5 post infection than on day 2 and more up-regulated differentially expressed microRNAs were provoked than the down-regulated for both strains of influenza virus. Finally, 47 differentially expressed microRNAs were obtained for the infection of both strains of H1N1 influenza virus with 29 for influenza virus BJ501 and 43 for PR8. Among them, 15 microRNAs had no reported function, while 32 including miR-155 and miR-233 are known to play important roles in cancer, immunity and antiviral activity. Pathway enrichment analyses of the predicted targets revealed that the transforming growth factor-β (TGF-β) signaling pathway was the key cellular pathway associated with the differentially expressed miRNAs during influenza virus PR8 or BJ501 infection. To our knowledge, this is the first report of microRNA expression profiles of the 2009 pandemic H1N1 influenza virus in a mouse model, and our findings might offer novel therapy targets for influenza virus infection.

## Introduction

Influenza A viruses infecting humans are responsible for a variety of illnesses ranging from mild infection to more severe pneumonia associated with acute respiratory distress syndrome. Even in non-pandemic years, influenza A viruses infect 5–15% of the global population and result in > 500,000 deaths annually [1]. In 2009, a novel strain of H1N1 influenza virus emerged in California and rapidly spread throughout the world [2]. A recent study estimated that > 284,000 deaths occurred globally during the first 12 months of 2009 pandemic H1N1virus circulation [3]. Given the possibility of reassortment of the 2009 pandemic H1N1 influenza virus, highly pathogenic H5N1 influenza viruses or co-circulating seasonal human H1N1 viruses, the threat posed by the 2009 pandemic H1N1 virus to humans remains significant [4,5]. Understanding the pathogenesis of influenza virus infection is essential to preventing and controlling future outbreaks.

MicroRNAs are 20-22 nucleotide length noncoding RNA molecules that act by repressing target protein expression at the post-transcriptional level. Mature microRNAs can specifically bind semi-complementarily to target mRNA, thereby triggering mRNA degradation or translation inhibition [6]. The human genome contains > 1,400 microRNA-coding genes, and > 60% of all human protein-coding genes are predicted to be microRNA targets. Functionally, microRNAs can target mRNA molecules involved in various biological processes, such as development, differentiation, proliferation, apoptosis and tumorigenesis [7,8,9]. Increasing evidence indicates that microRNAs have important functions in viral replication and may be used by host cells to inhibit or promote viral infections [10,11]. Expression of microRNAs has been reported for various viruses, such as human immunodeficiency virus [12], hepatitis B virus [13], hepatitis C virus [14] and Epstein-Barr virus [15].

Influenza virus infection has been shown to alter microRNA expression both in cultured cells and in animal models [16,17,18,19,20,21,22,23,24]. Using the microRNA microarray proﬁling approach, differentially expressed patterns of cellular microRNAs have been found in the lungs of mice infected with a highly pathogenic 1918 pandemic H1N1 influenza virus [16]. Another study found a strain-specific host microRNA signature associated with 2009 pandemic H1N1 and H7N7 influenza virus infections in human A549 cells [17]. In addition, differential microRNA expression profiles have been observed in the lungs of H5N1 influenza virus-infected cynomolgus macaques [18] and mice [19], H1N2 virus-infected pigs [21] and avian H5N3 influenza virus-infected broilers [20] and chickens [22]. All of these studies have provided strong evidence that microRNAs play an important role during influenza virus infection. Moreover, several studies have demonstrated that cellular microRNAs (miR-323, miR-491, miR654, miR-146a) inhibit influenza virus replication or propagation [23,24].

The mouse remains the primary model for studying the pathology and virulence of influenza virus [25]. However, there are no reports of the microRNA expression profile of the 2009 pandemic H1N1 influenza virus in a mouse model. In the present study, we successfully profiled the lung cellular microRNAs of mice infected with the 2009 pandemic influenza virus BJ501 and a comparison influenza virus PR8, and 29 microRNAs were found to be differentially expressed in response to influenza virus BJ501 infection compared to 43 to PR8; among them, 15 had no reported function in Pubmed, while 32 including miR-145, miR-155 and miR-233 were known to associate with cancer, immunity and antiviral activities. Some of the differentially expressed microRNAs might be potential therapeutic targets for influenza virus infection. 

## Materials and Methods

### Ethics statement

All procedures involving animals were approved by the Institute of Animal Care and Use Committee at AMMS. The animal study was carried out in strict accordance with the recommendations in the Guide for the Care and Use of Laboratory Animals of Beijing Institute of Disease Control and Prevention.

### Viruses

The virus strains used in this study included influenza virus A/Beijing/501/2009 (H1N1), an influenza virus isolated from a confirmed H1N1 influenza case in Beijing during 2009 [26,27], and A/Puerto Rico/8/34 (H1N1), a well characterized and mouse-adapted laboratory strain of influenza virus used as the genetic backbone for viruses from which inactivated influenza virus vaccines are generated. The A/Beijing/501/2009 H1N1 virus does not carry the D222G mutation reported in some H1N1 pandemic strains. The virus was grown in the allantoic cavities of 10-day-old embryonic chicken eggs. Virus-containing allantoic fluid was harvested and stored in aliquots at -80°C until use. The 50% tissue culture infections dose (TCID_50_) for each virus was determined by serial dilution of the virus in Madin-Darby canine kidney (MDCK) cells (ATCC, Virginia, USA) and calculated by the method developed by Reed and Muench [28]. All experiments with live influenza viruses were performed in an approved biosafety level 3 (BSL3+) facility.

### Viral infections in mice

Specific pathogen-free 6- to 8-week-old male BALB/c mice were provided by the Laboratory Animal Center, AMMS, Beijing, China. Inﬂuenza virus infections in mice were conducted as described previously [26]. Briefly, the mice were anesthetized with isoflurane and were intranasally inoculated with 10^4^ TCID_50_ (25 µL) of BJ501 or PR8 virus in phosphate buffered saline (PBS). Based on the pilot study, clinical signs, weight loss and lung damage were observed in mice infected with 10^4^ TCID_50_ of influenza virus BJ501 and PR8. In addition, a normal control group was given intranasal PBS (mock treatment).

### RNA isolation

For the total RNA extraction, entire lungs from mice infected by BJ501 or PR8 (n = 3/time-point) were randomly selected and harvested on 2 and 5 days post infection (dpi); entire lungs from 3 mock-infected mice were also randomly selected and harvested on 5 dpi. Whole mouse lung tissues were homogenized in QIAzol lysis reagent (Qiagen). Total RNA was extracted from mouse lungs using an mirVana microRNA Isolation Kit (Ambion) according to the manufacturer’s protocol. The concentration of RNA was determined using a Nanodrop ND-1000 Spectrophotometer (Thermo).

### MicroRNA microarray analysis

Three mice infected by BJ501 or PR8 on 2 and 5 dpi, and 3 mock-infected mice on 5 dpi were selected for microRNA microarray analysis. An Agilent Mouse microRNA v16.0 Microarray Kit (8×15K) was used according to the manufacturer’s instructions to profile the microRNA transcripts on an Agilent Technologies microRNA Platform (Santa Clara, CA). Briefly, 100 ng of a total RNA sample was used to make probes according to the manufacturer’s protocol. Probes were hybridized with rotation at 20 rpm for 20 h at 55°C. The array slides were then washed using gene expression wash buffer 1 at room temperature for 5 min and using gene expression wash buffer 2 at 37°C for 5 min. The slides were then scanned on an Agilent Microarray Scanner (Model #G2505C; Agilent). Microarray results were extracted using Agilent Feature Extraction software (v10.7). The differential expression of microRNAs between groups was assessed by one-way analysis of variance (ANOVA) with correction of Benjamini-Hochberg FDR applied and a Tukey’s honestly significant difference (HSD) post-hoc test. The expression change (fold-change) of a microRNA in a BJ501-infected sample or a PR8-infected sample relative to the mock-infected sample was calculated; the fold-change of an microRNA in a BJ501-infected sample compared to that in the time-matched PR8-infected sample was also calculated. Significance was determined using a fold-change threshold of at least 2 and a *P* value cut-off of 0.05. A fold change of 2 was chosen to improve the precise degree of results [17,29].

### Real-time reverse transcription polymerase chain reaction (RT-PCR)

Real-time RT-PCR was used to validate the microRNA microarray results. Total RNA was prepared using the mirVana microRNA Isolation Kit (Ambion) as described above. The cDNA was synthesized from mRNA with corresponding microRNA-specific stem-loop RT primers (Table 1) and M-MLV Reverse Transcriptase (Invitrogen). Real-time PCR was performed using Power SYBR Green PCR Master Mix (Applied Biosystems) with corresponding primers (Table 1) on a 7900 Real-Time PCR System (Applied Biosystems). The specificity of the SYBR Green PCR signal was confirmed by melting curve analysis. The PCR reaction mixture (20 µL) consisted of 10 µL of Power SYBR Green PCR Master Mix, 0.5 µL of microRNA-specific anti-sense primer (Invitrogen), 0.5 µL of universal sense primers, 1 µL of microRNA cDNA, and 8 µL of nuclease-free water. Cycling conditions were 95°C for 10 min followed by 40 cycles at 95°C for 15 sec and 60°C for 15 sec. U6 was used as endogenous control for normalization [20,30]. Data were analyzed using the 2^-△△Ct^ method [31].

**Table 1:** Primers for microRNAs in real-time RT-PCR analysis.

microRNAs	Primers sequences
U6	5'-CTCGCTTCGGCAGCACA-3' (sense)
	5'-AACGCTTCACGAATTTGCGT-3' (anti-sense)
miR-1	5'-GTCGTATCCAGTGCAGGGTCCGAGGTATTCGCACTGGATACGACAATACAT-3' (RT)
	5'-ACGCCTGGAATGTAAAGAAGTATG-3' (anti-sense)
miR-1187	5'-GTCGTATCCAGTGCAGGGTCCGAGGTATTCGCACTGGATACGACTTACAC-3' (RT)
	5'-AGCGTATGTGTGTGTGTATGTGTG-3' (anti-sense)
miR-133a	5'-GTCGTATCCAGTGCAGGGTCCGAGGTATTCGCACTGGATACGACCAGCTG-3' (RT)
	5'-ATCGGTCCCCTTCAACCAG-3' (anti-sense)
miR-133b	5'-GTCGTATCCAGTGCAGGGTCCGAGGTATTCGCACTGGATACGACTAGCTG-3' (RT)
	5'-GTTTGGTCCCCTTCAACCAG-3' (anti-sense)
miR-155	5'-GTCGTATCCAGTGCAGGGTCCGAGGTATTCGCACTGGATACGACACCCCT-3' (RT)
	5'-TGCGTTAATGCTAATTGTGATAGG-3' (anti-sense)
miR-2137	5'-GTCGTATCCAGTGCAGGGTCCGAGGTATTCGCACTGGATACGACCTCCCT-3' (RT)
	5'-GTGCTATGTGTGAGCCCCAG-3' (anti-sense)
miR-223	5'-GTCGTATCCAGTGCAGGGTCCGAGGTATTCGCACTGGATACGACTGGGGT-3' (RT)
	5'-ACGCTGTCAGTTTGTCAAATACC-3' (anti-sense)
miR-30d	5'-GTCGTATCCAGTGCAGGGTCCGAGGTATTCGCACTGGATACGACCTTCCA-3' (RT)
	5'-AGCTGTAAACATCCCCGACTG-3'(anti-sense)
miR-574-3p	5'-GTCGTATCCAGTGCAGGGTCCGAGGTATTCGCACTGGATACGACTGTGGG-3' (RT)
	5'-GTCACGCTCATGCACACACC-3' (anti-sense)

The sense primer of all the selected microRNAs are common, the sequence is 5'-GTGCAGGGTCCGAGGT-3'

### Bioinformatics analysis

The microRNA targets were predicted using the TargetScan version 6.2 database [32,33], which was downloaded from http://www.targetscan.org/. To more strictly predict the targets, the predicted microRNA targets found for the differentially expressed microRNAs in TargetScan version 6.2 database were selected by the cutoff of a context score percentile > 90.

The selected predicted targets underwent GO and pathway analysis using the functional annotation tools of DAVID 6.7 [34,35] (http://david.abcc.Ncifcrf.gov/home.jsp) with the mouse genome genes as the background set, and the enrichment of GO terms and pathways were selected with a cutoff standard of *P* < 0.01 and FDR < 1.

### Microarray data resource

The microarray data were deposited in the Gene Expression Omnibus database (www.ncbi.nlm.gov/geo/) under the accession number GSE46087. 

## Results

### Clinical manifestations and weight loss of mice infected with influenza virus BJ501 and PR8

To assess the virulence of influenza virus BJ501 and PR8 in mice, clinical signs and weight of the mice were monitored daily for 14 days. In the first dpi, no change in the general appearance was observed. However, decreased appetite and reduced activity were observed in both the BJ501 and PR8 infection groups from the 2 dpi following weight loss from the third dpi. The weight loss gradually increased to the 7 dpi and the weight began to reverse from the eighth dpi with gradual increase to the weight level of the control groups by 12 dpi. Moreover, compared with the influenza virus BJ501, the clinical signs, symptoms and weight loss caused by PR8 were more severe. No clinical signs and significant weight loss were observed in the mice of the control groups.

### MicroRNA expression pattern in response to influenza virus BJ501 and PR8 infection

**Figure 1:**
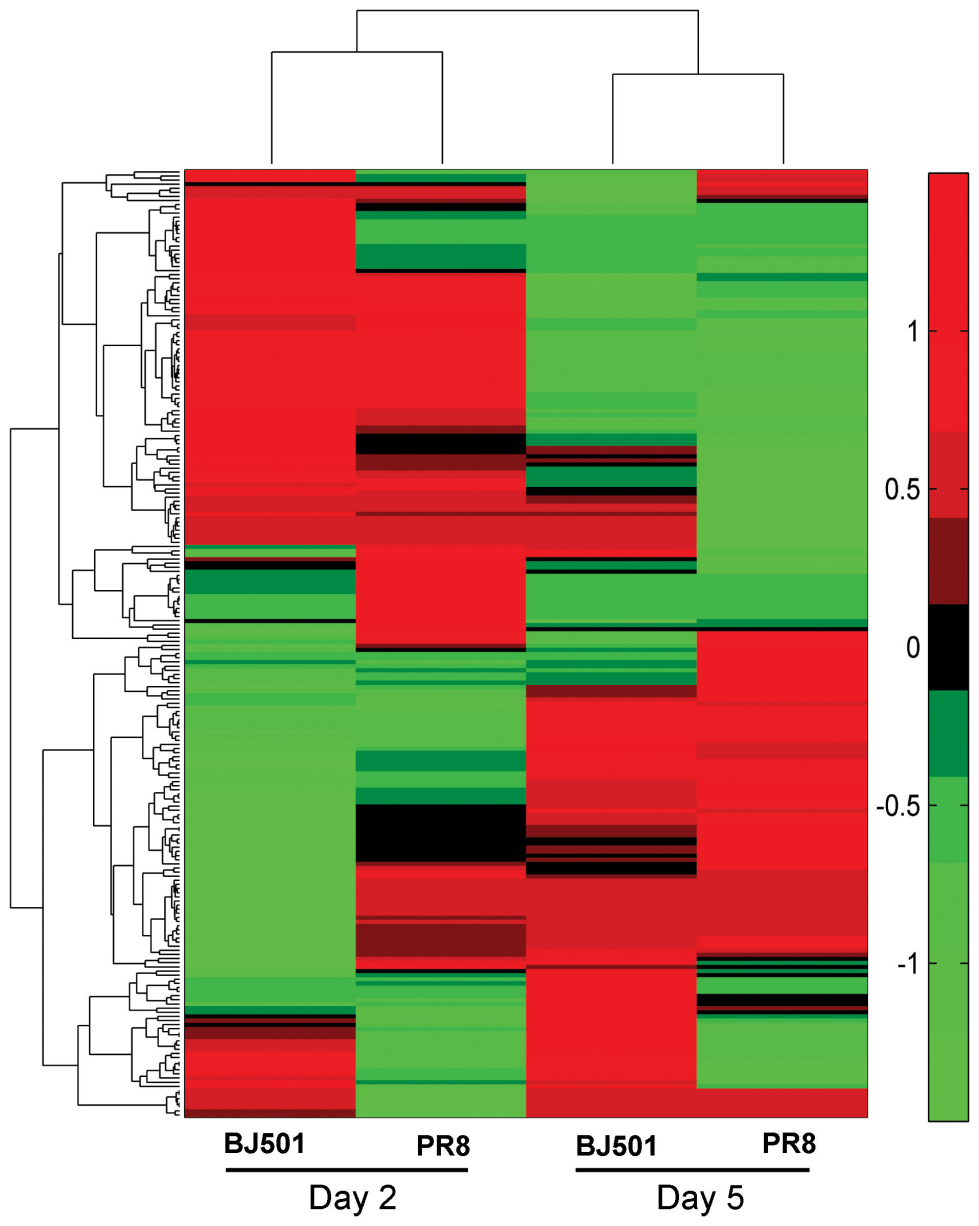
The microRNA expression patterns in response to influenza virus BJ501 and PR8 infections. The expression patterns of 230 detected microRNAs of influenza virus BJ501 and PR8 infections to the mock-infected controls on 2 dpi and 5 dpi were depicted with a clustered heatmap. The clustering tree is shown on the top and left sides. The red color represents up-regulation, while the green color indicates down-regulation.

To understand the effect of influenza virus infection on host microRNAs, we profiled the global expression of cellular microRNAs in response to influenza virus BJ501 and PR8 virus infection in a mouse model. Among 938 microRNAs on the arrays, 230 were detected in all of the mouse lungs and were included in a comparative analysis of microRNA expression patterns that were depicted using a clustered heatmap (Figure 1). The microRNA expression pattern induced by influenza virus BJ501 differed from that by influenza virus PR8 on 2 dpi and 5 dpi. The microRNA expression pattern induced by influenza virus BJ501 on 2 dpi differed from that by influenza virus BJ501 on 5 dpi and the same was with influenza virus PR8 in the temporal microRNA expression patterns (Figure 1). Therefore, the microRNA expression patterns induced by influenza virus BJ501 and PR8 were strain- and temporal-specific.

### Differentially expressed microRNAs in response to influenza virus BJ501 and PR8 infection

**Figure 2:**
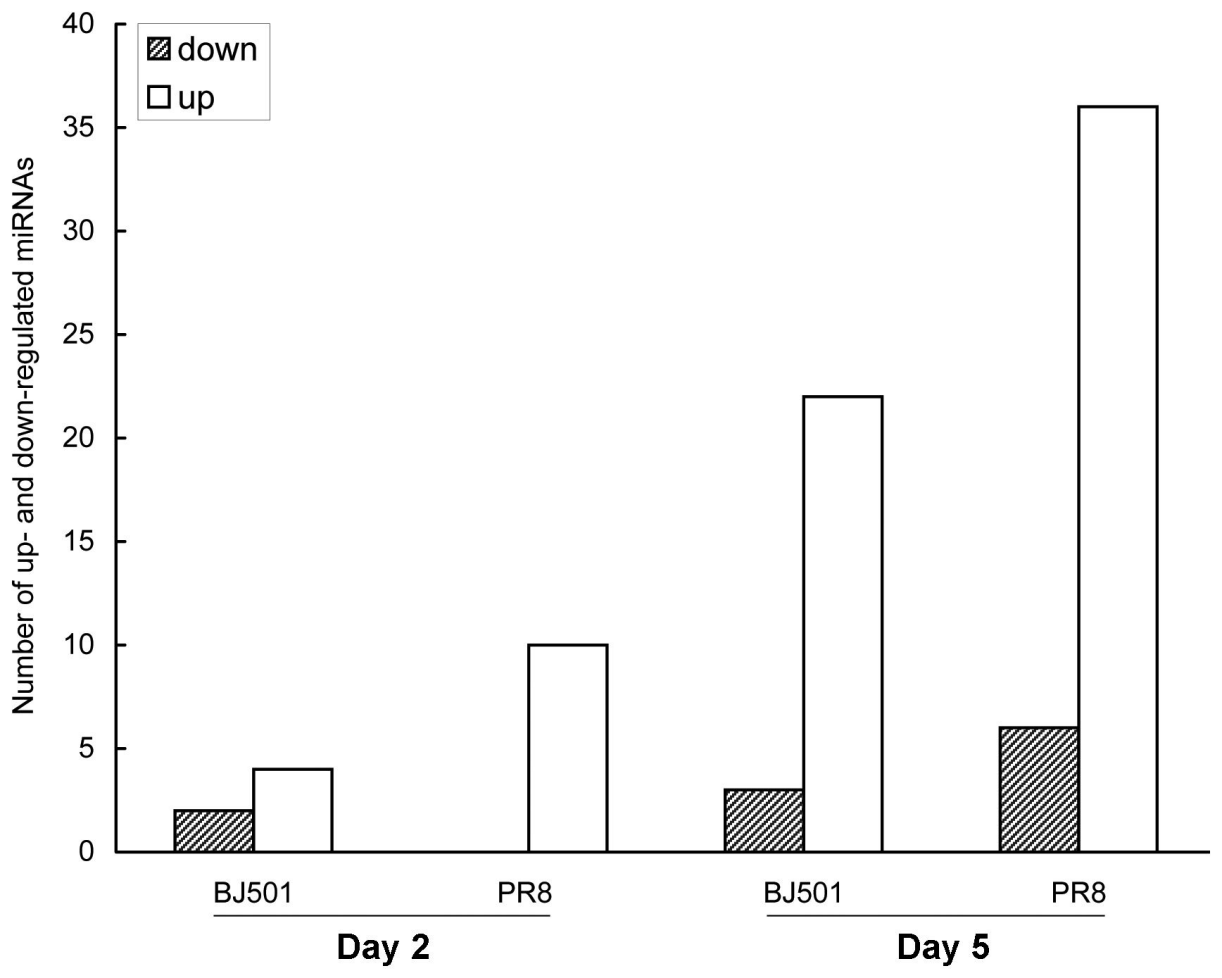
The number of differentially expressed microRNAs during influenza virus BJ501 and PR8 infections. The y axis indicates the number of differentially expressed microRNAs. Significance was determined using a fold-change threshold of at least 2 and a nominal *P* value cutoff of 0.05.

In influenza virus BJ501 infection, 6 microRNAs exhibited differential expression on 2 dpi; among them, 2 (33%) were down-regulated, while 4 (67%) were up-regulated (Figure 2). A total of 25 microRNAs were differentially expressed on 5 dpi, and the number of up-regulated microRNAs dramatically increased to 22 (88%) of the total differentially expressed microRNAs (Figure 2). It was important to note that except for mmu-miR-155 and mmu-miR-142-3p, the subset of differentially expressed microRNAs on 2 dpi was distinct from that on 5 dpi. Therefore, the number of differentially expressed microRNAs was 29 in all on 2 dpi and 5 dpi in response to influenza virus BJ501 infection.

In influenza virus PR8 infection, down-regulated microRNAs were not observed and 10 microRNAs were differentially up-regulated on 2 dpi (Figure 2). On 5 dpi, the number of differentially expressed microRNAs dramatically increased to 42; among them, 6 (10%) microRNAs were down-regulated and 38 (90%) microRNAs were up-

regulated (Figure 2). Interestingly, 9 of the differentially up-regulated microRNAs on 2 dpi were differentially expressed on 5 dpi as well, so the number of differentially expressed microRNAs was 43 in total on 2 dpi and 5 dpi in response to influenza virus PR8 infection.

**Figure 3:**
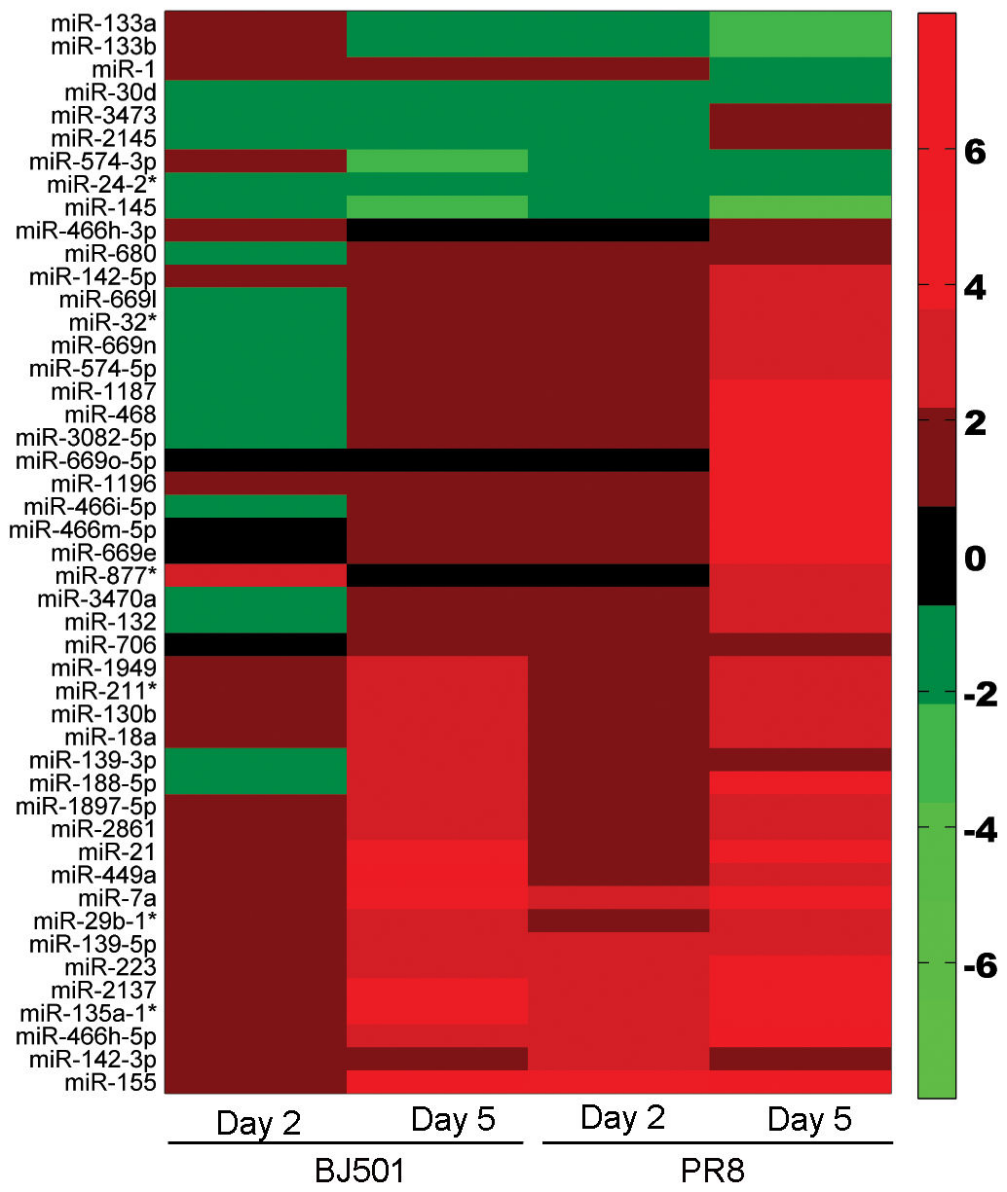
Distinct cellular microRNA expression patterns during influenza virus BJ501 and PR8 infections. The columns correspond to expression patterns of differentially expressed microRNAs during the influenza virus BJ501 and PR8 infections relative to mock-infected samples on 2 dpi and 5 dpi. Significance was determined using a fold-change threshold of at least 2 and a *P* value cutoff of 0.05. The red color represents up-regulation, while the green color indicates down-regulation.

In addition to the distinctly differentially expressed microRNAs in response to influenza virus BJ501 and PR8 infection, 8 microRNAs were common to PR8 on 2 dpi, BJ501 on 5 dpi and PR8 on 5 dpi and 15 microRNAs common to group of BJ501 on 5 dpi and PR8 on 5 dpi. Notably, mmu-miR-155 was commonly differentially expressed between both H1N1 strains at all time points. Therefore, the total number of differentially expressed microRNAs was 47 across all time points during influenza virus BJ501 and PR8 infection. The finding was depicted on a heatmap (Figure 3).

### Common and distinct differentially expressed microRNAs in response to influenza virus BJ501 and PR8 infection

**Figure 4:**
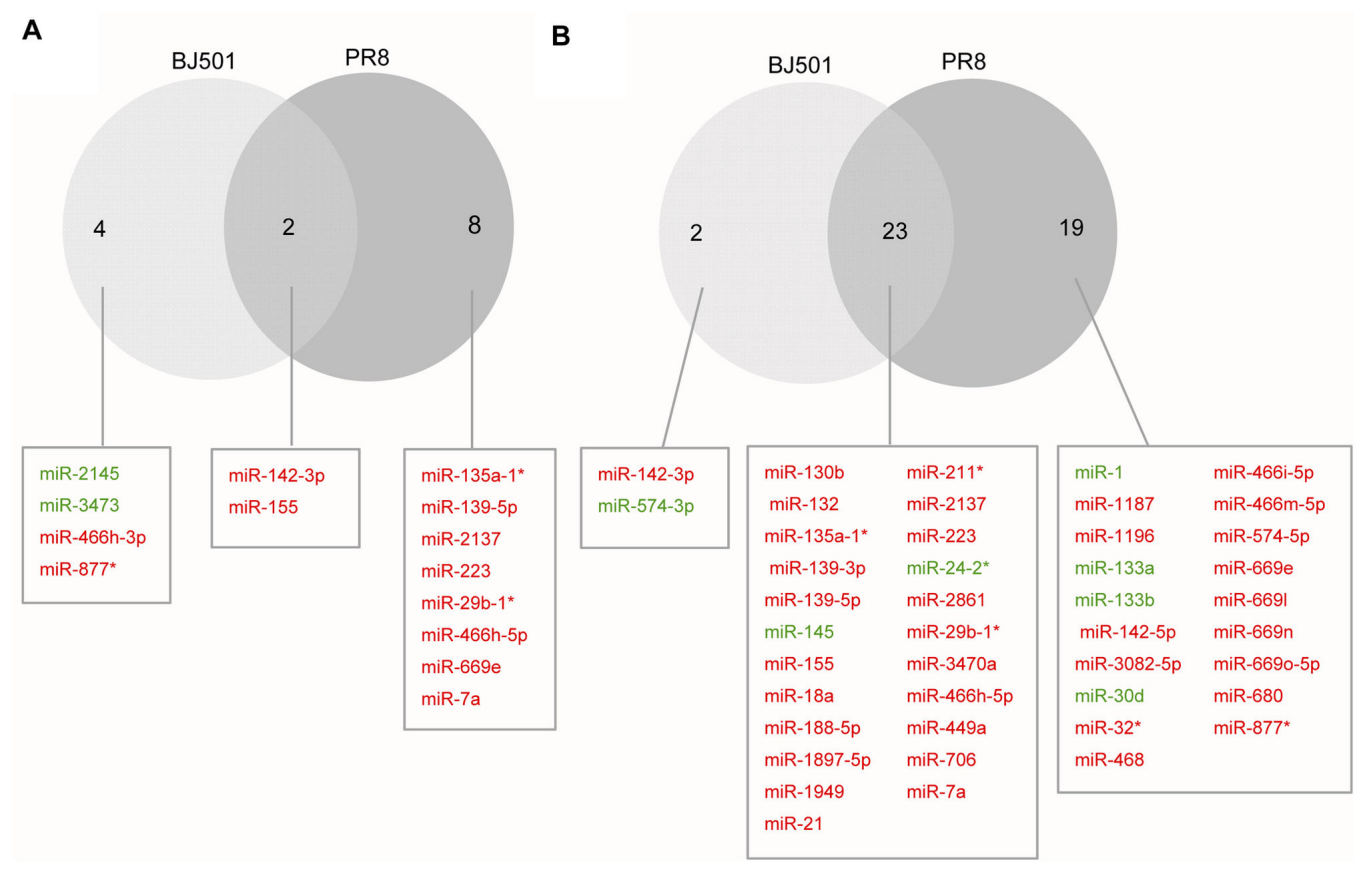
Comparison of differentially expressed microRNAs between the influenza virus BJ501 and PR8 infections. (A) Venn diagram of differentially expressed microRNAs during influenza virus BJ501 and PR8 infections relative to the mock-infected control on 2 dpi. (B) Venn diagram of differentially expressed microRNAs during influenza virus BJ501 and PR8 infections relative to the mock-infected control on 5 dpi. The diagram displays the names of differentially expressed microRNAs. The red color represents up-regulated microRNAs, while the green color indicates down-regulated microRNAs.

The differentially expressed microRNAs between influenza virus BJ501 and PR8 infections were compared. The common and distinct differentially expressed microRNAs between influenza virus BJ501- and PR8-infected lungs relative to mock-infected controls are shown in Figure 4. There were 12 distinct differentially expressed microRNAs on 2 dpi and 21 on 5 dpi. Of the common differentially expressed microRNAs, there were 2 on 2 dpi and 23 on 5 dpi. Therefore, the number of common and distinct differentially expressed microRNAs between the 2 strains increased over time.

The fold-change of microRNAs in influenza virus BJ501-infected samples on 2 dpi and 5 dpi compared to that in the time-matched influenza virus PR8-infected samples was also calculated (Table 2). Overall, we identified 17 microRNAs that were differentially expressed between the 2 strains. Eight microRNAs exhibited differential expression on 2 dpi and the number of differentially expressed microRNAs increased to 13 on 5 dpi, including 4 differentially expressed microRNAs on 2 dpi.

**Table 2:** Comparison of differentially expressed cellular microRNA between influenza virus BJ501 and PR8 infection.

microRNA	Fold change on 2 dpi^*^	Fold change on 5 dpi^*^
mmu-miR-466h-5p	-3.310513	-2.908281
mmu-miR-466i-5p	-2.742921	-2.309546
mmu-miR-468	-2.553224	-2.093672
mmu-miR-1187	-2.193327	-2.124729
mmu-miR-155	-2.265238	NS
mmu-miR-669l	-2.060334	NS
mmu-miR-574-5p	-2.032452	NS
mmu-miR-574-3p	2.223028	NS
mmu-miR-466m-5p	NS	-2.645127
mmu-miR-669e	NS	-2.633191
mmu-miR-1196	NS	-2.205374
mmu-miR-3082-5p	NS	-2.150287
mmu-miR-32*	NS	-2.118266
mmu-miR-133b	NS	2.090192
mmu-miR-133a	NS	2.159145
mmu-miR-449a	NS	2.313966
mmu-miR-1	NS	2.520204

Abbreviations: NS, Not Significant.

* The expression change (fold change) of a microRNA in influenza virus BJ501 infected sample compared to that in the time-matched influenza virus PR8 infected sample was calculated. Significance was determined by using a fold-change threshold of at least 2 and a nominal *P* value cut-off of 0.05.

### Verification of microRNA microarray results by real-time RT-PCR

**Figure 5:**
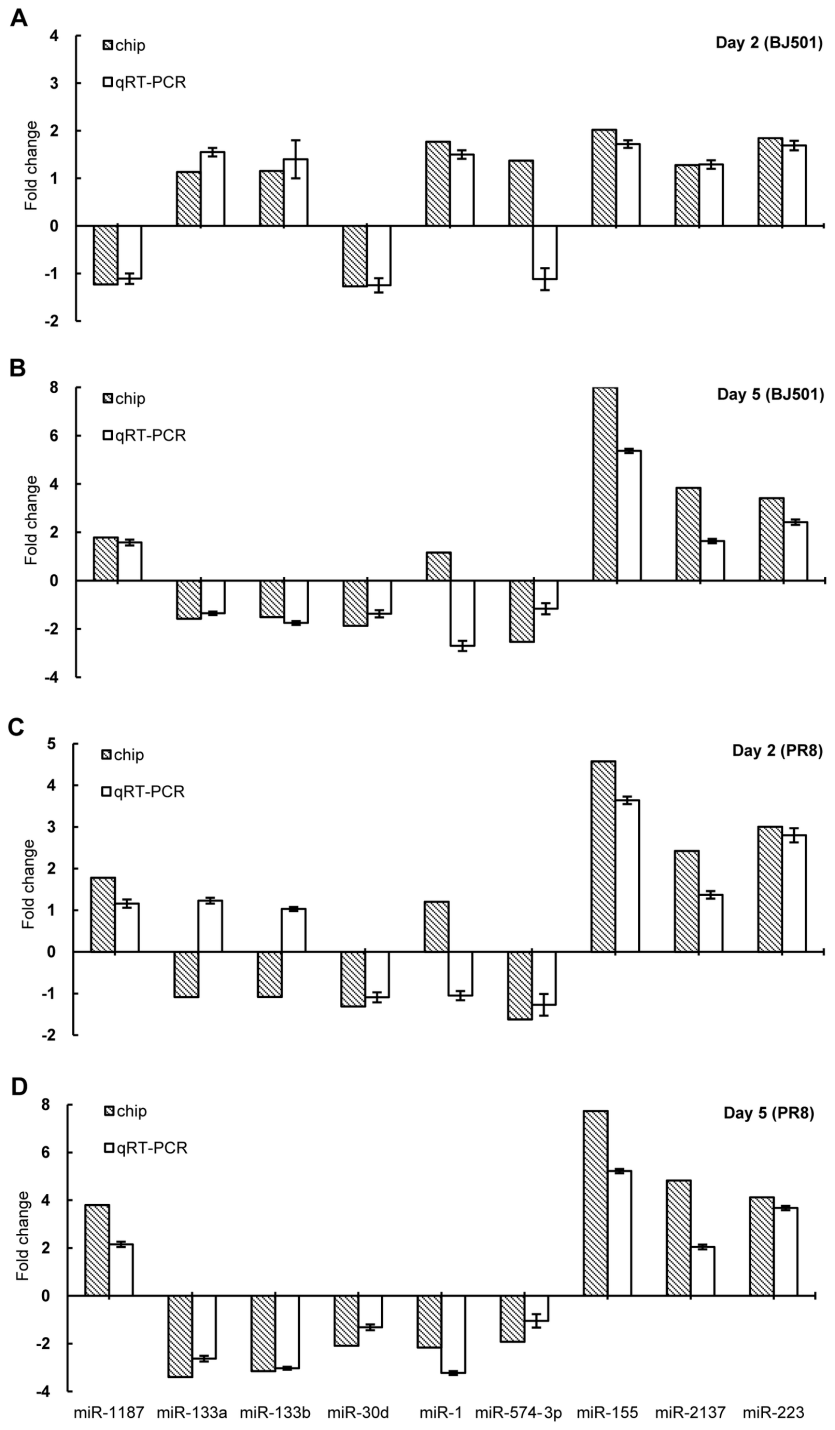
Real-time reverse transcription polymerase chain reaction (RT-PCR) verification of microRNA microarray. Nine differentially expressed microRNAs were selected from microarray datasets and examined by real-time RT-PCR. The fold change of a particular microRNA in the influenza virus BJ501 or PR8 infection relative to the mock infection was calculated. The fold-change from the real-time RT-PCR was determined using the 2-^△△Ct^ method and all microRNA expression values were normalized against the U6 endogenous control. Data from real-time RT-PCR are shown as mean ± standard deviation (SD).

Real-time RT-PCR was used to further investigate the microarray data set results. Nine microRNAs (miR-1, miR-1187, miR-133a, miR-133b, miR-155, miR-2137, miR-223, miR-30d and miR-574-3p) were selected for validation. The fold-change of a particular microRNA in the influenza virus BJ501 or PR8 infected lungs relative to the mock-infected lungs was calculated. We found that 86.11% (31 of 36) of the relative real-time RT-PCR results were consistent with those obtained in the microRNA microarray analysis in terms of direction of regulation at one or more time points except the results of miR-574-3p in BJ501-infected lung on 2 dpi, miR-1 in PR8-infected lung on 2 dpi, miR-1 in BJ501-infected lung on 5 dpi, miR-133a in PR8-infected lung on 2 dpi and miR-133b in PR8-infected lung on 2 dpi (Figure 5).

### GO analysis of predicted targets of differentially expressed microRNAs

**Figure 6:**
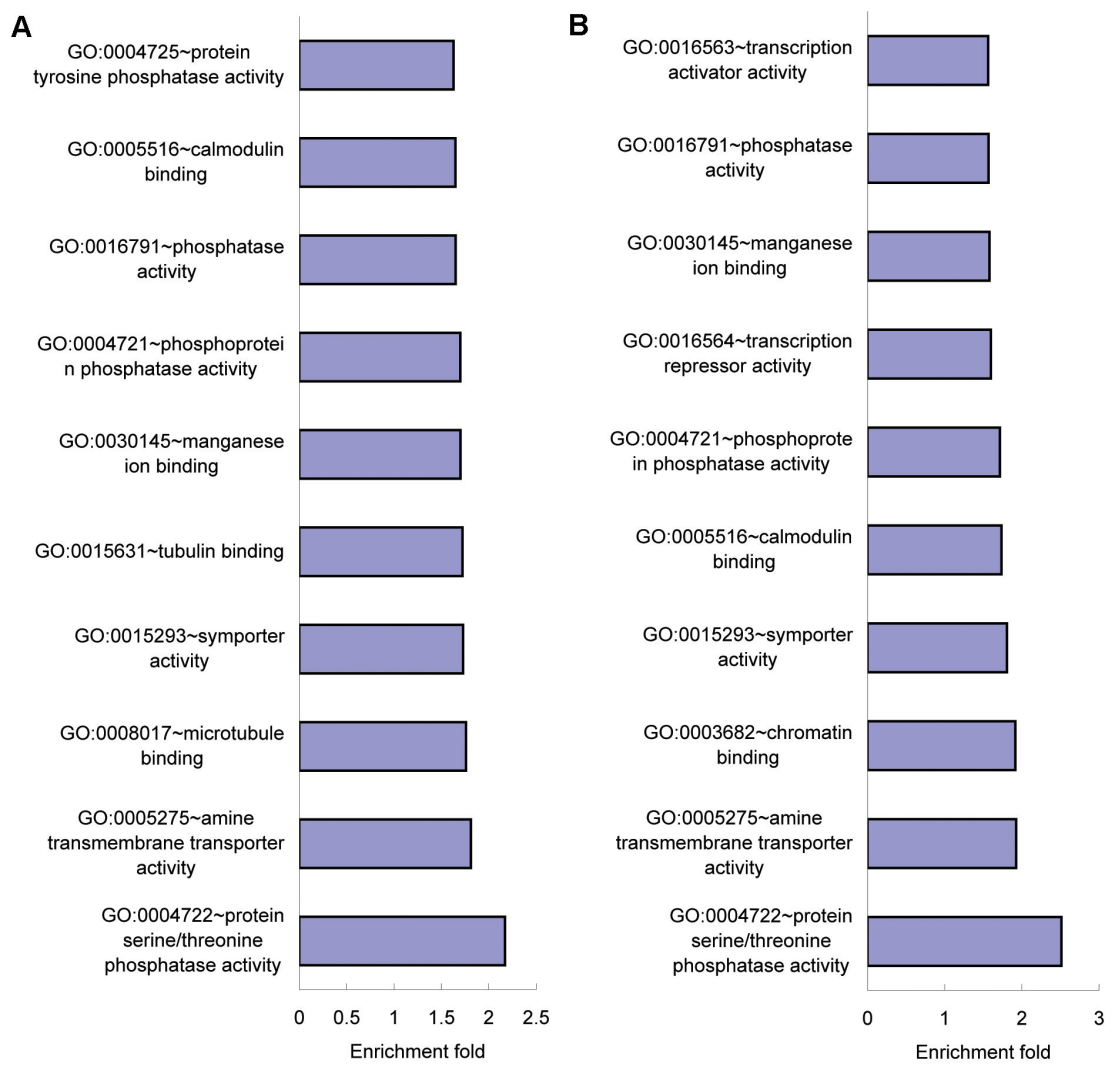
Top 10 significantly enriched GO terms of molecular function for the predicted targets. (A) Top ten significantly enriched GO terms of molecular function for the predicted targets of differentially expressed microRNAs in response to influenza virus BJ501 infection. (B) Top 10 significantly enriched GO terms of molecular function for the predicted targets of differentially expressed microRNAs in response to influenza virus PR8 infection.

To elucidate the roles of the differentially expressed microRNAs in response to influenza virus infection, potential targets of differentially expressed microRNAs were predicted using Targetscan 6.2 with a context score percentile > 90. There was no target for mmu-miR-1, mmu-miR-135a-1, mmu-miR-2145, mmu-miR-24-2 or mmu-miR-29b-1 in the database. In the influenza virus BJ501 infection, 4,033 predicted targets were obtained for the 29 differentially expressed microRNAs (Table S1). In the influenza virus PR8 infection, 5,446 predicted targets were obtained for the 43 differentially expressed microRNAs (Table S1). Then the 4,033 and 5,446 predicted targets were subjected to GO analysis in DAVID v6.7, respectively. After the cutoff standard of *P* < 0.01 and FDR < 1, 39 and 47 GO terms of molecular function were found to be involved in influenza virus BJ501 and PR8 infection, respectively (Table S2). Among the top 10 significantly enriched GO terms of molecular function, seven were common to the influenza virus BJ501 and PR8 infection: protein serine/threonine phosphatase activity, amine transmembrane transporter activity, symporter activity, manganese ion binding, phosphoprotein phosphatase activity, phosphatase activity and calmodulin binding (Figure 6).

### Kyoto Encyclopedia of Genes and Genomes (KEGG) pathway analysis of the predicted targets of differentially expressed microRNAs

**Figure 7:**
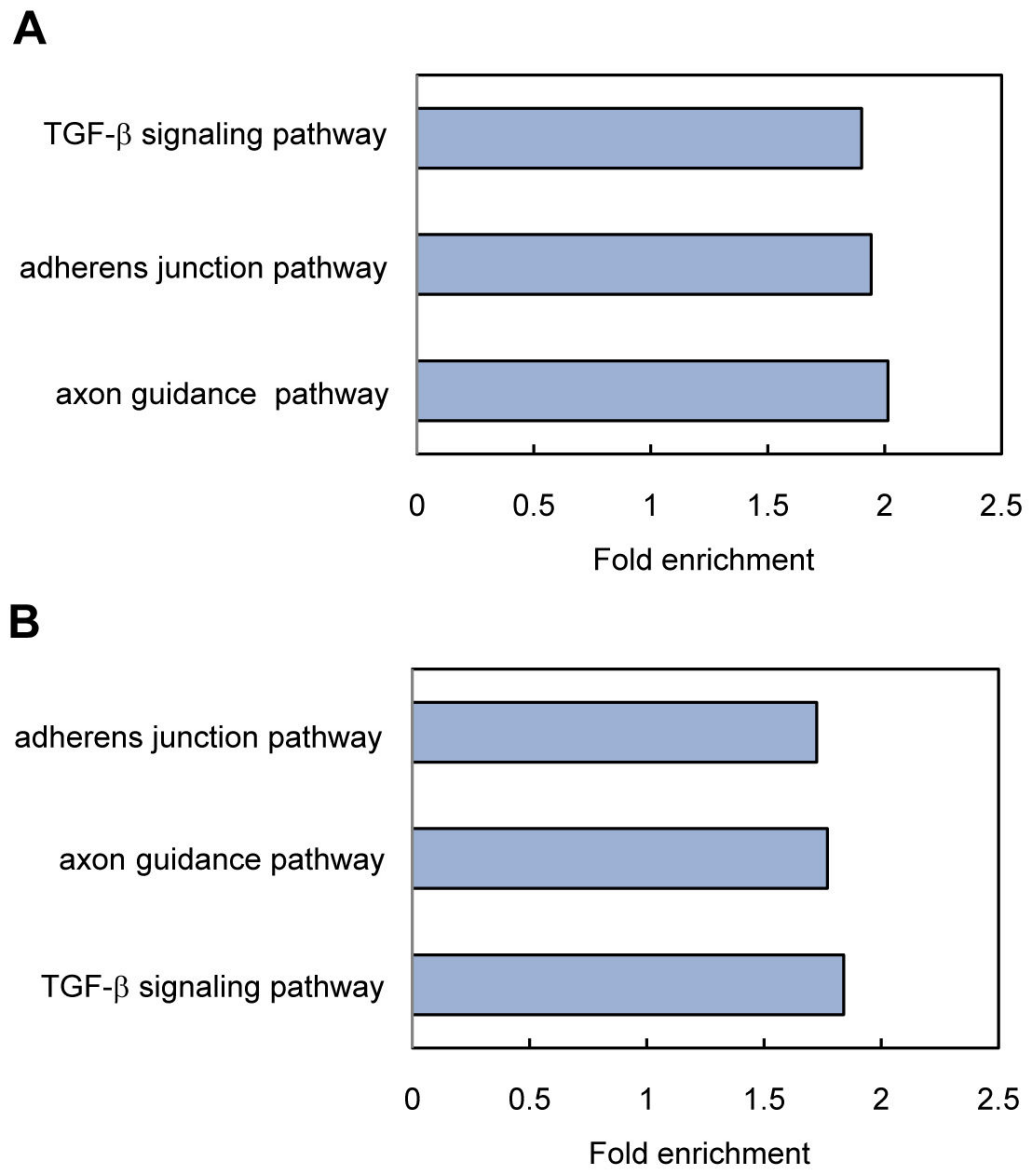
KEGG pathway analysis of the predicted targets of differentially expressed microRNAs. (A) The top three significantly enriched pathways in influenza virus BJ501 infection. (B) The top three significantly enriched pathways in influenza virus PR8 infection.

To better understand the roles of the differentially expressed microRNAs of influenza virus BJ501 and PR8 infection, their predicted targets genes were subjected to KEGG pathway enrichment analysis using DAVID 6.7. In the influenza virus BJ501 infection, 14 pathways were significantly enriched (Table S2). The top three were the transforming growth factor-β (TGF-β) signaling pathway, adherens junction and axon guidance pathway (Figure 7A). In the influenza virus PR8 infection, 10 pathways were significantly enriched (Table S2), and the top three were the adherens junction, axon guidance pathway and TGF-β signaling pathway (Figure 7B). Moreover, the 10 significantly enriched pathways of influenza virus PR8 infection were all enriched in influenza virus BJ501 infection as well: the TGF-β signaling pathway, adherens junction, axon guidance pathway, MAPK signaling pathway, focal adhesion, endocytosis, chemokine signaling pathway, regulation of actin cytoskeleton, ErbB signaling pathway and pathways in cancer (Table S2).

## Discussion

In this study, we found that strain- and temporal-specific microRNA expression patterns were induced by influenza virus infection. Specifically, a group of common and distinct microRNAs were differentially expressed in response to influenza virus BJ501 and PR8 infection; among them, 15 microRNAs had no reported function in Pubmed, while 32 had known functions that were mainly reported to be involved with cancer (Table S3). To our knowledge, this is the first report of microRNA expression profiles of the 2009 pandemic H1N1 influenza virus in mouse model.

The biological basis of the difference in disease severity and clinical outcome among different strains of influenza virus infections remains largely unknown. Herein we demonstrated the induction of a group of strain-specific host microRNAs in response to infection with H1N1 influenza virus PR8 and 2009 pandemic H1N1 influenza virus BJ501. In recent years, several studies have also reported strain-specific host microRNA expression profiles in response influenza virus infection. In 2010, Li et al. first reported that the reconstructed 1918 H1N1 virus and a seasonal H1N1 virus infection induced distinct microRNA expression profiles [16]. Subsequent works were carried out between the H5N1 virus and a 1918 H1N1 reassortant virus, and the findings further indicated that influenza viruses of varying pathogenicity elicit distinct microRNA expression patterns in a host response to infection [19]. Most recently, Loveday et al. found a series of strain-specific host microRNAs associated with the 2009 pandemic H1N1 influenza virus and H7N7 influenza virus infection in human A549 cells [17]. Our results, together with those of the earlier studies, suggest that different strains of influenza viruses elicit distinct microRNA expression patterns during infection. The potential of using these microRNAs as prognostic markers and therapeutic targets is worth being explored.

This is the second report aimed at elucidating the microRNA profile of 2009 pandemic H1N1 influenza virus infection. In the first report, Loveday et al. showed that a total of 52 microRNAs were signiﬁcantly modulated in human A549 cells upon infection with the 2009 pandemic H1N1 influenza virus [17]. In our study, 29 microRNAs were differentially expressed in response to 2009 pandemic H1N1 influenza virus infection, fewer than those of the Loveday et al. study. In addition, only miR-877* were common to both studies and had consistent direction of regulation following 2009 pandemic H1N1 influenza virus infection in two studies. These results indicate that a host’s genetic background may be an important factor affecting microRNA expression and regulation during infection with the 2009 pandemic H1N1 influenza virus. Interestingly, studies carried out in avian H5N3 influenza virus-infected lungs of broilers [20] and layer chickens [22] also indicate that genetic background is a critical factor in determining host cellular microRNA abundance and regulation during H5N3 virus infection. Thus, it is plausible to assume that host genetic background may play an important role in the regulation of microRNAs expression during influenza virus infection. However, these results warrant further confirmation in future studies.

The results of this microRNA profiling study provided unique insights into the pathogenesis of the mouse-adapted influenza virus PR8. Compared with BJ501, a higher number of differential microRNAs was aroused in the PR8 infected lungs and a group of microRNAs unique to PR8 were differentially expressed. Moreover, some differentially up-regulated microRNAs (such as miR-466h-5p, miR-135a-1*, miR-2137, miR-223, miR-139-5p, miR-29b-1*, and miR-7a) displayed earlier in PR8 infected lungs than in BJ501 infected lungs. It is well known that the mouse-adapted influenza virus showed increased virulence and pathogenecity in mice. Our results also showed that mice infected with PR8 showed more severe clinical signs, weight loss and lung damage than mice infected with BJ501. The potential molecular mechanisms underlying the high virulence of the adapted influenza virus were beyond the scope of this study, but our data suggested that the strain-specific differential microRNAs of PR8 observed here may be another important factor involved in its high virulence in mice.

A group of microRNAs including miR-155 and miR-223 have been identified to potentially regulate influenza virus infection in our study. miR-155 has been demonstrated to play an important role in the mammalian immune system. miR-155 knockout mice cannot generate defensive immune responses as a result of affected lymphocyte differentiation [36]. One study demonstrated that miR-155 might target the chicken anti-influenza gene *MX1* and activate the JUK pathway, therefore regulating influenza virus infection in chickens [22]. miR-223 is also an important microRNA with multiple functions [37]. Up-regulation of miR-223 has been reported in the lungs of mice infected with a highly pathogenic 1918 pandemic H1N1 influenza virus-infected lungs of mice [16], H5N1 virus-infected lungs of mice [19], and H1N2 virus-infected lungs of pigs [21]. In our study, miR-155 and miR-223 were significantly induced by both the 2009 pandemic H1N1 virus and the PR8 virus. Thus, it is plausible to assume that the miR-155 and miR-223 may play an important role in the response to influenza virus infection. However, this assumption warrants further confirmation in future studies.

It is well known that the TGF-β pathway participates in the processes of cell cycle control, differentiation and apoptosis and affects many pathophysiological responses, including the responses to viral infections [38,39]. Studies have shown that influenza virus can activate the TGF-β pathway both in vitro [40] and in vivo [39,41,42]. In a mouse model, the addition of TGF-β to influenza virus-infected mice reduced viral titers, whereas the neutralization of TGF-β increased morbidity [41]. In patients with 2009 pandemic H1N1 influenza virus infection, high levels of TGF-β were found in the plasma [42]. All these reports come to that the TGF-β pathway is implicated in the pathogenesis of influenza virus infection. Our pathway enrichment analysis of the microRNA-predicted targets showed the TGF-β pathway was enriched. Therefore, some of the differentially expressed microRNAs induced by the influenza virus BJ501 and PR8 may affect the pathophysiological process of influenza virus infection by regulating the TGF-β pathway.

In this study, we validated some differentially expressed microRNAs by real-time RT-PCR. The reliability of the profiling results was verified because the overall results of qualitative real-time were consistent with those of the microarray. However, five microRNA expression values at certain time point were opposite in direction between the results of the microarray and the RT-PCR. Among them, the differences were slight and not significant for four results of the microRNA. However, as for the expression of miR-1 in BJ501-infected lung on 5 dpi, it was not significantly up-regulated from the results of microarray analysis while it down-regulated > 2-fold by RT-PCR analysis. The possible factors leading to the difference were multiple and complex for different sensitivity and specificity between the two methods, so further confirmation were warranted in future studies.

In reviewing the results of this study, two potential limitations should be kept in mind. First, analyzing the correlation of microRNA and mRNA profiles from the same pathogen-infected sample may increase our understanding of microRNA function in response to influenza virus infection. Second, the identification of differentially expressed host cellular microRNAs is just the first step toward understanding microRNA regulation of host–virus interactions. Although this study generated a list of candidate microRNAs including miR-155 and miR-223 that potentially regulate influenza virus infections, additional studies are warranted to clarify the mechanisms behind how these candidate microRNAs mediate host–virus interactions during influenza virus infection.

In conclusion, here we identified a group of strain-specific and common cellular microRNAs that were associated with influenza virus BJ501 and PR8 infections. Further microRNA or gene-specific knockdown experiments are necessary to elucidate the underlying mechanism of microRNAs in response to influenza virus infection. These findings might offer novel targets for developing new therapies against influenza virus infection. 

## Supporting Information

Table S1 **Targets of the differential expressed miRNA predicted from Targetscan 6.2 database.**
(XLS)

Table S2 **GO and KEGG enrichment analysis of the targets of the differential expressed miRNAs.**
(XLS)

Table S3 **Differential expressed miRNAs of known and unknown functions.**
(XLS)

## References

[1] Stöhr K (2002) Influenza--WHO cares. Lancet Infect Dis 2: 517. doi:10.1016/S1473-3099(02)00366-3. PubMed: 12206966. 12206966 10.1016/s1473-3099(02)00366-3

[2] Smith GJ, Vijaykrishna D, Bahl J, Lycett SJ, Worobey M et al. (2009) Origins and evolutionary genomics of the 2009 swine-origin H1N1 influenza A epidemic. Nature 459: 1122-1125. doi:10.1038/nature08182. PubMed: 19516283. 19516283 10.1038/nature08182

[3] Dawood FS, Iuliano AD, Reed C, Meltzer MI, Shay DK et al. (2012) Estimated global mortality associated with the first 12 months of 2009 pandemic influenza A H1N1 virus circulation: a modelling study. Lancet Infect Dis 12: 687-695. doi:10.1016/S1473-3099(12)70121-4. PubMed: 22738893. 22738893 10.1016/S1473-3099(12)70121-4

[4] Itoh Y, Shinya K, Kiso M, Watanabe T, Sakoda Y et al. (2009) In vitro and in vivo characterization of new swine-origin H1N1 influenza viruses. Nature 460: 1021-1025. PubMed: 19672242. 19672242 10.1038/nature08260 PMC2748827

[5] Cline TD, Karlsson EA, Freiden P, Seufzer BJ, Rehg JE et al. (2011) Increased pathogenicity of a reassortant 2009 pandemic H1N1 influenza virus containing an H5N1 hemagglutinin. J Virol 85: 12262-12270. doi:10.1128/JVI.05582-11. PubMed: 21917948. 21917948 10.1128/JVI.05582-11 PMC3209346

[6] Bartel DP (2004) MicroRNAs: genomics, biogenesis, mechanism, and function. Cell 116: 281-297. doi:10.1016/S0092-8674(04)00045-5. PubMed: 14744438. 14744438 10.1016/s0092-8674(04)00045-5

[7] Ambros V (2004) The functions of animal microRNAs. Nature 431: 350-355. doi:10.1038/nature02871. PubMed: 15372042. 15372042 10.1038/nature02871

[8] Lu J, Getz G, Miska EA, Alvarez-Saavedra E, Lamb J et al. (2005) MicroRNA expression profiles classify human cancers. Nature 435: 834-838. doi:10.1038/nature03702. PubMed: 15944708. 15944708 10.1038/nature03702

[9] Bartel DP (2009) MicroRNAs: target recognition and regulatory functions. Cell 136: 215-233. doi:10.1016/j.cell.2009.01.002. PubMed: 19167326. 19167326 10.1016/j.cell.2009.01.002 PMC3794896

[10] Grassmann R, Jeang KT (2008) The roles of microRNAs in mammalian virus infection. Biochim Biophys Acta 1779: 706-711. doi:10.1016/j.bbagrm.2008.05.005. PubMed: 18549828. 18549828 10.1016/j.bbagrm.2008.05.005 PMC2641032

[11] Ghosh Z, Mallick B, Chakrabarti J (2009) Cellular versus viral microRNAs in host-virus interaction. Nucleic Acids Res 37: 1035-1048. PubMed: 19095692. 19095692 10.1093/nar/gkn1004 PMC2651794

[12] Houzet L, Yeung ML, de Lame V, Desai D, Smith SM et al. (2008) MicroRNA profile changes in human immunodeficiency virus type 1 (HIV-1) seropositive individuals. Retrovirology 5: 118. doi:10.1186/1742-4690-5-118. PubMed: 19114009. 19114009 10.1186/1742-4690-5-118 PMC2644721

[13] Pan XB, Ma H, Jin Q, Wei L (2012) Characterization of microRNA expression profiles associated with hepatitis B virus replication and clearance in vivo and in vitro. J Gastroenterol Hepatol 27: 805-812. doi:10.1111/j.1440-1746.2011.06979.x. PubMed: 22097931. 22097931 10.1111/j.1440-1746.2011.06979.x

[14] Liu X, Wang T, Wakita T, Yang W (2010) Systematic identification of microRNA and messenger RNA profiles in hepatitis C virus-infected human hepatoma cells. Virology 398: 57-67. doi:10.1016/j.virol.2009.11.036. PubMed: 20006370. 20006370 10.1016/j.virol.2009.11.036

[15] Imig J, Motsch N, Zhu JY, Barth S, Okoniewski M et al. (2011) microRNA profiling in Epstein-Barr virus-associated B-cell lymphoma. Nucleic Acids Res 39: 1880-1893. doi:10.1093/nar/gkq1043. PubMed: 21062812. 21062812 10.1093/nar/gkq1043 PMC3061055

[16] Li Y, Chan EY, Li J, Ni C, Peng X et al. (2010) MicroRNA expression and virulence in pandemic influenza virus-infected mice. J Virol 84: 3023-3032. doi:10.1128/JVI.02203-09. PubMed: 20071585. 20071585 10.1128/JVI.02203-09 PMC2826040

[17] Loveday EK, Svinti V, Diederich S, Pasick J, Jean F (2012) Temporal- and strain-specific host microRNA molecular signatures associated with swine-origin H1N1 and avian-origin H7N7 influenza A virus infection. J Virol 86: 6109-6122. doi:10.1128/JVI.06892-11. PubMed: 22438559. 22438559 10.1128/JVI.06892-11 PMC3372180

[18] Li Y, Li J, Belisle S, Baskin CR, Tumpey TM et al. (2011) Differential microRNA expression and virulence of avian, 1918 reassortant, and reconstructed 1918 influenza A viruses. Virology 421: 105-113. doi:10.1016/j.virol.2011.09.011. PubMed: 21999992. 21999992 10.1016/j.virol.2011.09.011 PMC3210927

[19] Rogers JV, Price JA, Wendling MQ, Long JP, Bresler HS (2012) Preliminary microRNA analysis in lung tissue to identify potential therapeutic targets against H5N1 infection. Viral Immunol 25: 3-11. PubMed: 22233254. 22233254 10.1089/vim.2011.0047

[20] Wang Y, Brahmakshatriya V, Lupiani B, Reddy SM, Soibam B et al. (2012) Integrated analysis of microRNA expression and mRNA transcriptome in lungs of avian influenza virus infected broilers. BMC Genomics 13: 278. doi:10.1186/1471-2164-13-278. PubMed: 22726614. 22726614 10.1186/1471-2164-13-278 PMC3496578

[21] Skovgaard K, Cirera S, Vasby D, Podolska A, Breum SO et al. (2013) Expression of innate immune genes, proteins and microRNAs in lung tissue of pigs infected experimentally with influenza virus (H1N2). Innate Immun. 10.1177/1753425912473668 23405029

[22] Wang Y, Brahmakshatriya V, Zhu H, Lupiani B, Reddy SM et al. (2009) Identification of differentially expressed miRNAs in chicken lung and trachea with avian influenza virus infection by a deep sequencing approach. BMC Genomics 10: 512. doi:10.1186/1471-2164-10-512. PubMed: 19891781. 19891781 10.1186/1471-2164-10-512 PMC2777939

[23] Song L, Liu H, Gao S, Jiang W, Huang W (2010) Cellular microRNAs inhibit replication of the H1N1 influenza A virus in infected cells. J Virol 84: 8849-8860. doi:10.1128/JVI.00456-10. PubMed: 20554777. 20554777 10.1128/JVI.00456-10 PMC2919005

[24] Terrier O, Textoris J, Carron C, Marcel V, Bourdon JC et al. (2013) Host microRNA molecular signatures associated with human H1N1 and H3N2 influenza A viruses reveal an unanticipated antiviral activity for miR-146a. J Gen Virol 94: 985-995. doi:10.1099/vir.0.049528-0. PubMed: 23343627. 23343627 10.1099/vir.0.049528-0

[25] Barnard DL (2009) Animal models for the study of influenza pathogenesis and therapy. Antiviral Res 82: A110-A122. doi:10.1016/j.antiviral.2009.02.190. PubMed: 19176218. 19176218 10.1016/j.antiviral.2008.12.014 PMC2700745

[26] Sun S, Zhao G, Xiao W, Hu J, Guo Y et al. (2011) Age-related sensitivity and pathological differences in infections by 2009 pandemic influenza A (H1N1) virus. Virol J 8: 52. doi:10.1186/1743-422X-8-52. PubMed: 21299904. 21299904 10.1186/1743-422X-8-52 PMC3041774

[27] Yang P, Deng J, Li C, Zhang P, Xing L et al. (2012) Characterization of the 2009 pandemic A/Beijing/501/2009 H1N1 influenza strain in human airway epithelial cells and ferrets. PLOS ONE 7: e46184. doi:10.1371/journal.pone.0046184. PubMed: 23049974. 23049974 10.1371/journal.pone.0046184 PMC3458874

[28] Neumann G, Kawaoka Y (2001) Reverse genetics of influenza virus. Virology 287: 243-250. doi:10.1006/viro.2001.1008. PubMed: 11531402. 11531402 10.1006/viro.2001.1008

[29] Yeung ML, Bennasser Y, Myers TG, Jiang G, Benkirane M et al. (2005) Changes in microRNA expression profiles in HIV-1-transfected human cells. Retrovirology 2: 81. doi:10.1186/1742-4690-2-S1-S81. PubMed: 16381609. 16381609 10.1186/1742-4690-2-81 PMC1352379

[30] Zhao P, Zhao L, Zhang T, Wang H, Qin C et al. (2012) Changes in microRNA expression induced by rabies virus infection in mouse brains. Microb Pathog 52: 47-54. doi:10.1016/j.micpath.2011.10.001. PubMed: 22015383. 22015383 10.1016/j.micpath.2011.10.001

[31] Livak KJ, Schmittgen TD (2001) Analysis of relative gene expression data using real-time quantitative PCR and the 2(-Delta Delta C(T)) Method. Methods 25: 402-408. doi:10.1006/meth.2001.1262. PubMed: 11846609. 11846609 10.1006/meth.2001.1262

[32] Grimson A, Farh KK, Johnston WK, Garrett-Engele P, Lim LP et al. (2007) MicroRNA targeting specificity in mammals: determinants beyond seed pairing. Mol Cell 27: 91-105. doi:10.1016/j.molcel.2007.06.017. PubMed: 17612493. 17612493 10.1016/j.molcel.2007.06.017 PMC3800283

[33] Lewis BP, Burge CB, Bartel DP (2005) Conserved seed pairing, often flanked by adenosines, indicates that thousands of human genes are microRNA targets. Cell 120: 15-20. doi:10.1016/j.cell.2004.12.035. PubMed: 15652477. 15652477 10.1016/j.cell.2004.12.035

[34] Huang da W, Sherman BT, Lempicki RA (2009) Systematic and integrative analysis of large gene lists using DAVID bioinformatics resources. Nat Protoc 4: 44-57. PubMed: 19131956. 19131956 10.1038/nprot.2008.211

[35] Huang da W, Sherman BT, Lempicki RA (2009) Bioinformatics enrichment tools: paths toward the comprehensive functional analysis of large gene lists. Nucleic Acids Res 37: 1-13. doi:10.1093/nar/gkp505. PubMed: 19033363. 19033363 10.1093/nar/gkn923 PMC2615629

[36] Thai TH, Calado DP, Casola S, Ansel KM, Xiao C et al. (2007) Regulation of the germinal center response by microRNA-155. Science 316: 604-608. doi:10.1126/science.1141229. PubMed: 17463289. 17463289 10.1126/science.1141229

[37] Rangrez AY, M’Baya-Moutoula E, Metzinger-Le Meuth V, Henaut L, Djelouat MS et al. (2012) Inorganic phosphate accelerates the migration of vascular smooth muscle cells: evidence for the involvement of miR-223. PLOS ONE 7: e47807. doi:10.1371/journal.pone.0047807. PubMed: 23094093. 23094093 10.1371/journal.pone.0047807 PMC3475714

[38] Jones KS, Akel S, Petrow-Sadowski C, Huang Y, Bertolette DC et al. (2005) Induction of human T cell leukemia virus type I receptors on quiescent naive T lymphocytes by TGF-beta. J Immunol 174: 4262-4270. PubMed: 15778389. 15778389 10.4049/jimmunol.174.7.4262

[39] Schultz-Cherry S, Hinshaw VS (1996) Influenza virus neuraminidase activates latent transforming growth factor beta. J Virol 70: 8624-8629. PubMed: 8970987. 8970987 10.1128/jvi.70.12.8624-8629.1996 PMC190955

[40] Lam WY, Yeung AC, Chu IM, Chan PK (2010) Profiles of cytokine and chemokine gene expression in human pulmonary epithelial cells induced by human and avian influenza viruses. Virol J 7: 344. doi:10.1186/1743-422X-7-344. PubMed: 21108843. 21108843 10.1186/1743-422X-7-344 PMC3002310

[41] Carlson CM, Turpin EA, Moser LA, O’Brien KB, Cline TD et al. (2010) Transforming growth factor-beta: activation by neuraminidase and role in highly pathogenic H5N1 influenza pathogenesis. PLOS Pathog 6: e1001136. 20949074 10.1371/journal.ppat.1001136 PMC2951376

[42] Wen Y, Deng BC, Zhou Y, Wang Y, Cui W et al. (2011) Immunological features in patients with pneumonitis due to influenza A H1N1 infection. J Investig Allergol Clin Immunol 21: 44-50. PubMed: 21370723. 21370723

